# Qualitative and Quantitative Metabolite Comparison of Grain, Persimmon, and Apple Vinegars with Antioxidant Activities

**DOI:** 10.3390/antiox14081029

**Published:** 2025-08-21

**Authors:** Hyun-Ji Tak, Sowon Yang, So-Young Kim, Na-Rae Lee, Choong Hwan Lee

**Affiliations:** 1 Department of Bioscience and Biotechnology, Konkuk University, Seoul 05029, Republic of Korea; cathytak@konkuk.ac.kr (H.-J.T.); ysw6575@naver.com (S.Y.); 2 Department of Agrofood Resources, National Institute of Agricultural Sciences, Rural Development Administration, Wanju 55365, Republic of Korea; foodksy@korea.kr; 3 Research Institute for Bioactive-Metabolome Network, Konkuk University, Seoul 05029, Republic of Korea

**Keywords:** fermented vinegar, non-targeted metabolome, targeted metabolome, non-volatile metabolite, volatile metabolite

## Abstract

Fermented vinegars have been highlighted globally for their health benefits. The benefits can differ according to the type of vinegar; therefore, we investigated the differences of 15 grain (GV), 10 persimmon (PV), and 14 apple vinegars (AV) using integrated non-targeted and targeted metabolome analyses. We profiled non-volatile and volatile metabolites using gas chromatography time-of-flight mass spectrometry (GC-TOF-MS), ultra-high-performance liquid chromatography–orbitrap–tandem mass spectrometry, and headspace–solid-phase microextraction–GC-TOF-MS. Among the 132 identified metabolites, 73 non-volatile and 40 volatile metabolites showed significant differences across the three vinegar types. Amino acids, hydroxy fatty acids, phenolic compounds, aldehydes, pyrazines, and sulfides were abundant in GV. Some phenolic compounds, alcohols, and esters were abundant in PV, whereas carbohydrates, flavonoids, and terpenoids were abundant in AV, contributing to nutrients, tastes, and flavors. Bioactivity assays revealed that GV showed notable antioxidant activity, whereas PV and AV had the highest total phenolic and flavonoid contents, respectively. Through quantitative analysis, we revealed that acetic acid, propionic acid, butanoic acid, lactic acid, and alanine were major components in the three types of vinegar, although their composition was different in each vinegar. Our comprehensive qualitative and quantitative metabolite comparison provides insights into the differences among the three vinegar types, classified according to their raw materials.

## 1. Introduction

Vinegar, a popular food condiment, has a history of more than 3000 years [1]. It is produced from various raw materials including fruits (e.g., apples, persimmons, grapes, plums, and cherries) and cereals (e.g., rice, sorghum, barley, malt, wheat, corn, rye, and oats) [2,3,4]. Owing to the different raw materials, fermented vinegars have different flavors and nutrients [2,5].

**Figure 1:**
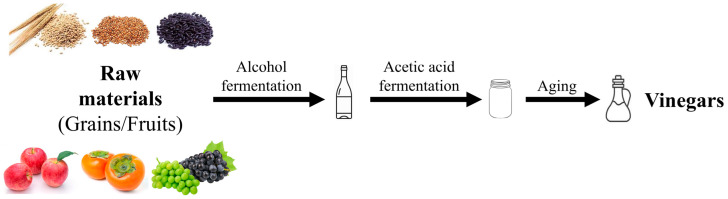
Manufacturing process of fermented vinegar products using various ingredients.

Vinegars are currently receiving considerable attention owing to their potential health benefits (e.g., anti-obesity, cholesterol-lowering, antimicrobial, antioxidant, antidiabetic, antitumor, and antihypertensive effects) [6,7,8,9]. Organic acids, amino acids, flavonoids, and phenolic compounds in vinegars, generated during the fermentation process or originating from raw materials, have been reported to have beneficial health effects [2,10]. For example, apple cider vinegar helps to reduce blood sugar content and blood pressure. Moreover, acetic acid and polyphenols in vinegars give antimicrobial and antioxidant properties. Vinegar production involves three main stages: ethanol fermentation, acetic acid fermentation, and aging (Figure 1). Ethanol and acetic acid fermentation are mainly conducted by yeasts (e.g., *Saccharomyces cerevisiae*) and acetic acid bacteria (e.g., *Acetobacter aceti*, *Acetobacter cerevisiae*, and *Gluconacetobacter oxydans*), respectively [11,12,13,14]. While producing acetic acid, other metabolites such as organic acids, amino acids, and fatty acids, which influence taste and health, are also generated from the raw materials [5,15,16,17]. Different raw materials result in dissimilar metabolite compositions in each type of vinegar, contributing to distinct health benefits [7,8,9,18,19,20].

Metabolomics is an analytical tool used to characterize the metabolite compositions of specimens, microbes, and plants, etc. [21,22,23]. This approach has also been applied in food research to reveal metabolite differences in food ingredients and changes during food processing, storage, and fermentation, etc. [24,25,26]. So far, the metabolome of numerous fermented foods, such as yogurt, cheese, and alcohol, has been used for analysis [27,28,29]. There have also been several metabolite analyses of vinegars [30]. Most studies that have analyzed vinegars have performed targeted metabolite analyses using a limited number of vinegars to compare the contents of well-known metabolites, such as organic acids, amino acids, and phenolic compounds, which can only provide nutritional information [2,20,31]. However, using only targeted metabolomic approach can mislead researchers because it can miss important metabolites while focusing on the targeted metabolites such as flavors. To comprehensively understand the organoleptic and nutritional properties of vinegars, it is necessary to conduct an untargeted metabolite analysis of non-volatile and volatile compounds [32].

In this study, we conducted non-targeted metabolite analyses using gas chromatography time-of-flight mass spectrometry (GC-TOF-MS), ultra-high-performance liquid chromatography–orbitrap–tandem mass spectrometry (UHPLC-Orbitrap-MS/MS), and headspace–solid-phase microextraction–GC-TOF-MS (HS-SPME-GC-TOF-MS) on various vinegars to comprehensively understand their metabolite contents. We selected 39 commercial vinegar products fermented from grains, persimmons, and apples in Korea—where persimmon vinegar is a unique traditional product, while apple and grain vinegars are more commonly available in other countries—to investigate metabolomic differences based on raw materials. Subsequently, based on the non-targeted metabolite profiling results and previous studies, we listed and quantified the organic acids, amino acids, and fatty acids of each product. To evaluate the potential health benefits of the three different vinegars, their antioxidant activities, total flavonoid content (TFC), and phenolic content (TPC) were also analyzed. Thus, we can provide qualitative and quantitative metabolomic information regarding various vinegars, which helps to understand the characteristics of vinegars based on raw materials.

## 2. Materials and Methods

### 2.1. Chemicals and Reagents

All chemicals and reagents were purchased from Fisher Scientific (Pittsburgh, PA, USA), Junsei Chemical Co., Ltd. (Tokyo, Japan), and Sigma-Aldrich (St. Louis, MO, USA).

### 2.2. Vinegar Samples

Thirty-nine vinegars produced from seven different provinces of South Korea were purchased. The vinegars were sorted by main raw materials (15 grain vinegars (GV), 10 persimmon vinegars (PV), and 14 apple vinegars (AV)). Detailed information such as sample number, pH, total acidity, province of manufacture, and ingredients is described in Table S1 for all products.

### 2.3. Non-Targeted Metabolite Analysis

Individual and pooled samples for each type of vinegar by combining 1 mL of each product were prepared to evaluate the metabolomic differences among the three types of vinegar. For metabolite extraction, 450 µL of methanol containing internal standards (2-chloro-L-phenylalanine, 1 mg/mL in water) was added to 50 µL of each sample or pooled samples, and then the mixture was vortexed and sonicated for 5 min. The extracted solution was filtered and dried using 0.2 μm polytetrafluoroethylene (PTFE) syringe filters (Chromdisc, Daegu, Republic of Korea) and a speed vacuum concentrator (Biotron, Seoul, Republic of Korea), respectively. The dried extracts were re-dissolved in methanol to make 5 mg/mL solution. The extracts of the individual and pooled samples were analyzed by respective GC-TOF-MS and UHPLC-Orbitrap-MS/MS. To analyze the volatile compounds in the vinegars, pooled samples of each type of vinegar were used for HS-SPME-GC-TOF-MS analysis.

#### 2.3.1. GC-TOF-MS Metabolite Analysis

The metabolite analysis method for GC-TOF-MS followed that of a previous study, with some modifications [29]. Re-dissolved extract (100 µL) was aliquoted and dried for derivatization. The dried sample was treated with 50 μL of methoxyamine hydrochloride (20 mg/mL in pyridine) and incubated at 30 °C for 90 min. Following this, 50 μL of MSTFA (*N*-methyl-*N*-(trimethylsilyl)trifluoroacetamide) was added for silylation, and the mixture was reacted at 37 °C for 30 min. The derivatized samples were filtered through a 0.2 μm PTFE syringe filter before injection. A 1 μL was injected into the GC-TOF-MS system at a split ratio of 15:1. The analysis was conducted using an Agilent 7890 B GC system coupled with a Pegasus BT TOF-MS (LECO). Chromatographic separation employed an Rtx-5MS capillary column (30 m × 0.25 mm × 0.25 μm, Restek Corp., Bellefonte, PA, USA). Helium was used as the carrier gas at a flow rate of 1.0 mL/min. The oven temperature was initially set to 75 °C for 2 min, then ramped at 15 °C/min to 300 °C, which was maintained for 3 min. The detector voltage was 2065 V, with a mass scan range of 50–600 *m*/*z*. The inlet and ion source temperatures were both set to 250 °C. All experiments were performed in triplicate.

#### 2.3.2. UHPLC-Orbitrap-MS/MS Metabolite Analysis

Using a previously established method with some modifications [29], UHPLC-Orbitrap-MS/MS analysis was conducted. All pooled extract samples of each vinegar type were reconstituted in methanol at a concentration of 0.5 mg/mL, and then filtered through a 0.2 μm PTFE syringe filter prior to injection. The analysis was performed on a Vanquish UHPLC system (Thermo Fisher Scientific, Waltham, MA, USA), equipped with a binary pump, autosampler, and column oven. Separation was achieved with an ACQUITY UPLC HSS T3 column (100 Å, 100 mm × 2.1 mm × 1.8 µm particle size; Waters Corp., Milford, MA, USA) using a gradient of mobile phases containing 0.1% formic acid in water (A) and 0.1% formic acid in acetonitrile (B). The program started with 5% B for 1 min, then ramped linearly to 100% B over 9 min, held for 1 min, and then reverted to initial conditions over 2 min. The flow rate was 0.3 mL/min, and 5 μL of sample was injected. Data acquisition was performed within the 100–1500 *m*/*z* range on an Orbitrap Exploris TM 120 mass spectrometer (Thermo Fisher Scientific). All analyses were conducted in triplicate to ensure data reliability.

#### 2.3.3. HS-SPME-GC-TOF-MS Metabolite Analysis

The analytical method for volatile organic compounds (VOCs) was used as described by Al-Dalali et al. with some modifications [33]. To extract VOCs from vinegar samples, 8 mL of pooled sample for each type of vinegar was added in the 20 mL SPME vial with 1.9 g of sodium chloride and 10 μL of internal solution (1,2-dichlorobenzene, 50 mg/L in methanol). Each sample was continuously stirred at 250 rpm and 45 °C to allow the reaction to reach equilibrium for 5 min. Fifty/thirty micrometers of divinylbenzene/carboxen/polydimethylsiloxane fiber (Sigma-Aldrich, St. Louis, MO, USA) were injected into the SPME vial and VOCs were extracted to the headspace of sample supernatants for 30 min at 45 °C. Then, the VOCs were detached to the GC-TOF-MS instrument (7890A GC system coupled with a Pegasus HT TOF-MS (LECO); Agilent Technologies, Santa Clara, CA, USA) for 5 min in splitless mode. Chromatographic separation was achieved using an Rtx-5MS capillary column (Restek Corp., Bellefonte, PA, USA) with helium as the carrier gas at a 1.0 mL/min flow rate. The GC column oven temperature was initially set at 40 °C for 3.5 min and increased to 90 °C at a rate of 5 °C/min. After reaching 90 °C, the temperature was ramped up to 230 °C at a rate of 12 °C/min and held for 12 min. The detector voltage was 1630 V, and the mass scan range was 30–450 *m*/*z*. The temperature of the inlet line and ion source were both set at 250 °C. All experiments were performed in triplicates.

### 2.4. Determination of Antioxidant Activities and Total Flavonoid and Phenolic Content

The antioxidant activity of the vinegar samples was assessed through ABTS (2,2′-azino-bis(3-ethylbenzothiazoline-6-sulfonic acid)), FRAP (Ferric Reducing Antioxidant Power), TFC (total flavonoid content), and TPC (total phenolic content) assays, using pooled extracts from each vinegar category. The protocols were adapted from a previous study with slight modifications, and each analysis was performed in triplicate for consistency [29].

For the ABTS assay, a 7 mM solution was prepared by dissolving 38.4 mg of ABTS in 10 mL of a potassium persulfate (2.45 mM) solution. After incubation at 60 °C for 20 min, the solution was stored at 4 °C overnight. Before analysis, the solution was diluted with water until reaching an absorbance of 0.7 ± 0.02 at 750 nm. Mixing 10 μL of the sample extract (5000 ppm) with 190 μL of the diluted ABTS solution in a 96-well plate, the reaction was incubated for 7 min at room temperature in the dark, and absorbance was measured at 750 nm.

The FRAP assay was executed by combining a mixture of acetate buffer (300 mM, pH 3.6), TPTZ (2,4,6-tris(2-pyridyl)-s-triazine, 10 mM in HCl), and FeCl_3_ (20 mM) in a 10:1:1 ratio. A 10 μL aliquot of sample extract (5000 ppm) was added to 300 μL of FRAP reagent and incubated for 6 min at room temperature. Absorbance was then recorded at 570 nm. The results of the antioxidant assays (ABTS and FRAP) were represented as the Trolox (6-hydroxy-2,5,7,8-tetramethylchroman-2-carboxylic acid) equivalent antioxidant capacity concentration (mM). The standard curves were generated over a concentration range from 0.015625 to 1 mM.

For TFC, 20 μL of extract was mixed with 20 μL of 1 N NaOH and 180 μL of 90% diethylene glycol, and the mixture was incubated in the dark for 60 min at room temperature. The TPC was determined by adding 20 μL of extract (5000 ppm) to 100 μL of Folin-Ciocalteu reagent, followed by 5 min incubation, and then 80 μL of Na_2_CO_3_ was added and incubated for 60 min. Absorbance readings for TFC and TPC were taken at 405 nm and 750 nm, respectively. Results were standardized against naringin and gallic acid calibration curves. The standard curves ranged from 1.5625 to 100 mg/L for naringin and from 3.90625 to 500 mg/L for gallic acid. All obtained values from antioxidant activity assays were normalized to the raw material volume.

### 2.5. Metabolite Quantification Analysis

#### 2.5.1. Quantification of Organic Acids

For the quantification of organic acids, an ultra-high-performance liquid chromatography–diode array detector (UHPLC-DAD) was used. Vinegar samples were first diluted with water, and then 5 μL of a levulinic acid internal standard solution (4 mg/mL in water) was added [34]. The mixture was filtered through a 0.2 μm PTFE syringe filter prior to instrument injection. An UHPLC system was equipped with a Vanquish binary pump F system, Vanquish Split Sampler FT, and DIONEX ULTIMATE 3000 RS DAD (Thermo Fisher Scientific). Chromatographic separation was achieved on an ACQUITY UPLC BEH C18 column (150 mm × 2.1 mm × 1.7 µm particle size; Waters Corp., Milford, MA, USA) at 30 °C. The mobile phase consisted of 0.02 mol/L sulfuric acid solution in water, with a flow rate of 0.1 mL/min and 2 μL injection volume. Absorbance was monitored at 210 nm [34]. All measurements were performed in triplicate. Standard calibration curves were prepared within the concentration ranges listed in Table S2. The limits of detection (LOD) and quantification (LOQ) were calculated using the standard deviation of the response (σ) and slope of the calibration curve (S) by using the formula LOD = 3.3 σ/S and LOQ = 10 σ/S.

#### 2.5.2. Quantification of Amino Acids

For amino acid quantification, a high-performance liquid chromatography with a diode array detector (HPLC-DAD) was used, following a slightly modified protocol from an earlier study [35,36]. First, equal amounts of OPA solution (20 mg/mL in 0.4 N borate buffer) and 3-MPA solution (20 mg/mL in 0.4 N borate buffer) were mixed. To each 4 μL of vinegar sample, 4 μL of the OPA/3-MPA mixture and 0.1 mg/mL L-norvaline in 0.1 M HCl were added. After 1 min, 20 μL of 0.4 N borate buffer and 4 μL of FMOC-Cl solution (2.5 mg/mL in acetonitrile) were added, followed by dilution with 84 μL water. The mixture was filtered through a 0.2 μm PTFE syringe filter prior to analysis. Separation was carried out on a HITACHI Chromaster chromatograph (HITACHI, Tokyo, Japan) using a Zorbax Eclipse-AAA column (4.6 × 150 mm × 5 µm particle size with guard column; Agilent Technologies) at 40 °C. The injection volume was 2 µL. Mobile phase A was 0.04 mol/L sodium dihydrogen phosphate (NaH_2_PO_4_), adjusted to pH 7.8 with 10 N sodium hydroxide (NaOH) solution. Mobile phase B comprised acetonitrile, methanol, and water (45:45:10, *v*/*v*/*v*). The gradient conditions (vol. %) were designed as follows: 0 min, 0% B; 2.9 min, 0% B; 19.1 min, 57% B; 19.6 min, 100% B; 23.3 min, 100% B; 24.2 min, 0% B; and 30 min, 0% B. The flow rate was set to 2 mL/min. Detection occurred at 262 nm for proline and 330 nm for other amino acids. All samples were analyzed in triplicate, and calibration curves, LOD, and LOQ were calculated accordingly.

#### 2.5.3. Quantification of Fatty Acids

For the quantification of fatty acids, an UHPLC-Triple Quadrupole Tandem Mass Spectrometry (UHPLC-Triple Q-MS/MS) system was employed. The derivatization protocol was adapted from earlier studies [37,38]. To prepare samples, 50 μL of each vinegar sample was mixed with 10 μL of internal standard solution (4-methyl valeric acid, 0.01 mg/mL). Then, 20 μL of 0.2 M 3-NPH solution (3-Nitrophenylhydrazine, 37.92 mg/mL in water) and 20 μL of 0.12 mM EDC solution (1-Ethyl-3-(3-dimethylaminopropyl) carbodiimide hydrochloride, 23.004 mg/mL in water containing 6% pyridine) were added to the sample mixture. The mixture was incubated at 40 °C for 30 min to allow for derivatization. After incubation, acetonitrile (200 μL) including 1% formic acid was added and filtered using a 0.2 μm PTFE syringe filter before instrumental analysis. The analysis was performed on a Shimadzu LC30AD system (Shimadzu Corp., Kyoto, Japan) coupled with a triple quadrupole MS/MS and an electrospray source (LCMS-8040, Shimadzu Corp.). MS data were collected in the range of 100–500 *m*/*z*. The chromatographic separation was conducted using Acquity UPLC BEH C18 column (100 mm × 2.1 mm × 1.7 μm particle size; Waters Corp.) at 30 °C. The mobile phase consisted of water with 1% formic acid (A) and acetonitrile with 1% formic acid (B). The gradient conditions (vol. %) were designed as follows: 0 min, 15% B; 2 min, 15% B; 30 min, 40% B; 36 min, 100% B; 38 min, 100% B; 38.5 min, 15% B; and 40 min, 15% B. The flow rate was 0.35 mL/min and 5 µL of each sample was injected. Triplicate analyses were performed, and the standard curves, LODs, and LOQs were established as described previously.

### 2.6. Data Processing and Multivariate Statistical Analysis

For GC-TOF-MS and HS-SPME-GC-TOF-MS data, files were converted to NetCDF (*. cdf) files using the LECO ChromaTOF software (version 4.44). MetAlign software (version 1.0.0.1; http://www.metalign.nl; accessed on 22 July 2024) was utilized for peak detection, retention time collection, and alignment. Raw UHPLC-Orbitrap-MS/MS data were converted to mzXML files using the ProteoWizard (version 3.0.23222-88f2e68; https://proteowizard.sourceforge.io/, accessed on 22 July 2024), and then further converted to ABF files using the Reifycs Analysis Base File Converter (https://www.reifycs.com/abfconverter/; accessed on 22 July 2024). MS-DIAL (version 5.1.230807; https://systemsomicslab.github.io/compms/msdial/main.html, accessed on 22 July 2024) was harnessed for peak picking, retention time correction, and alignment. Tentative metabolite identification was achieved by comparing mass retention times, fragment patterns, and spectral patterns with standard compounds and databases, such as the National Institutes of Standards and Technology Library (NIST; version 2.0, 2011; FairCom, Gaithersburg, MD, USA), PubChem (https://pubchem.ncbi.nlm.nih.gov/, accessed on 22 July 2024), and the Human Metabolome Database (HMDB; https://hmdb.ca/, accessed on 22 July 2024). Multivariate statistical analyses, including principal component analysis (PCA) and partial least squares discriminant analysis (PLS-DA), were performed using the SIMCA-P + (version 15.0.2; Umetrics, Umea, Sweden). Discriminant metabolites were selected based on variable importance in the projection (VIP) > 0.7 from the PLS-DA score plots, and significant differences (*p*-value < 0.05) were assessed by one-way analysis of variance (ANOVA) using STATISCA (version 7.0, StaSoft Inc., Tulsa, OK, USA).

## 3. Results and Discussion

### 3.1. Non-Targeted Metabolite Profiling of Grain, Persimmon, and Apple Vinegars

**Figure 2:**
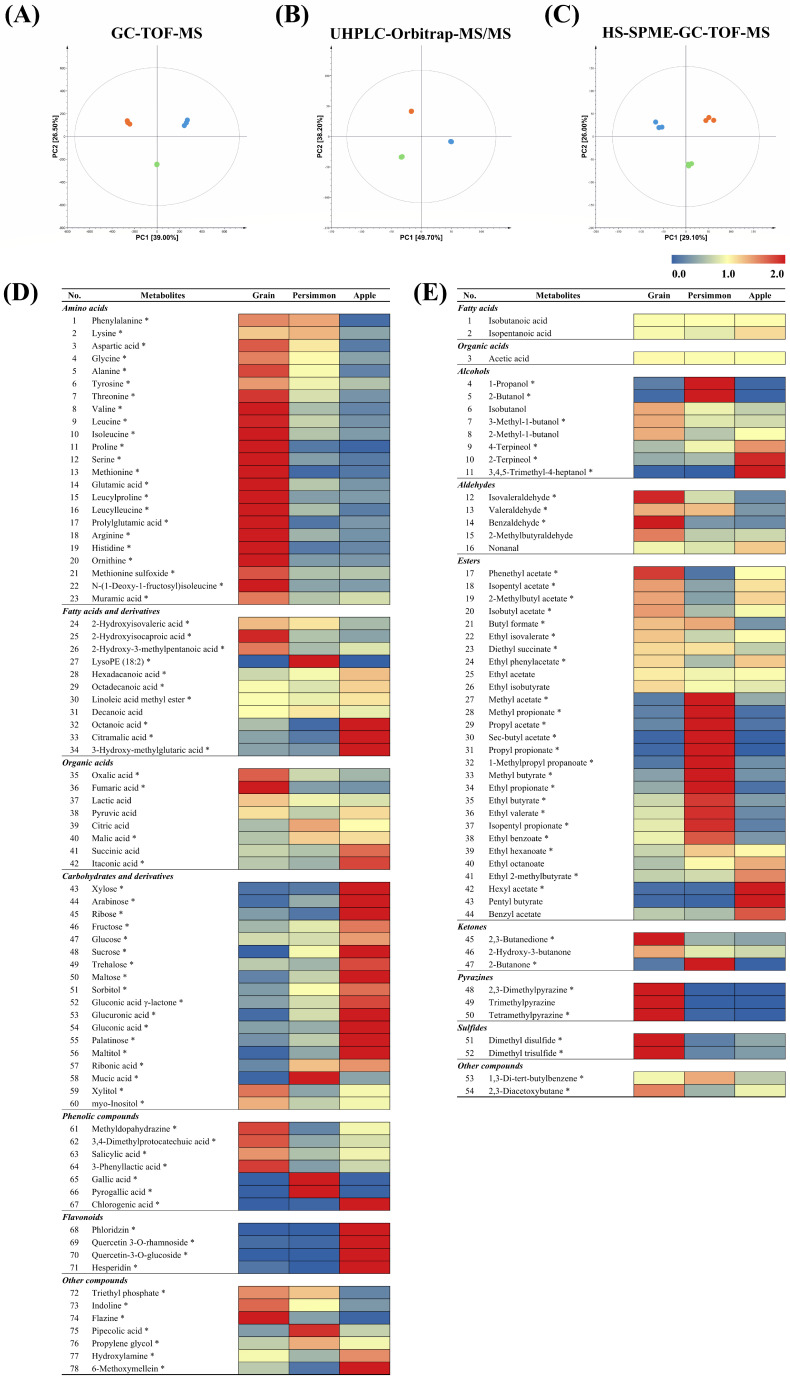
Principal component analysis (PCA) score plot of GV (●), PV (●), and AV (●) based on the GC-TOF-MS (**A**), UHPLC-Orbitrap-MS/MS (**B**), and HS-SPME-GC-TOF-MS (**C**) analysis datasets. All experiments were performed in triplicate. Heatmap of non-volatile and volatile metabolites based on three types of vinegars using GC-TOF-MS and UHPLC-Orbitrap-MS/MS (**D**) and HS-SPME-GC-TOF-MS (**E**) analyses, respectively. The heatmap based on the PLS-DA model highlights the relative abundance of observed metabolites. * The asterisks indicate the significant difference (VIP score > 0.7, *p*-value < 0.05).

To determine the metabolites in GV, PV, and AV, we conducted non-targeted metabolite profiling of non-volatile and volatile metabolites in combination with GC-TOF-MS, UHPLC-Orbitrap-MS/MS, and HS-SPME-GC-TOF-MS analyses using pooled samples of each type of vinegar. We identified 132 metabolites (78 non-volatile metabolites and 54 volatile metabolites), of which 113 metabolites (73 non-volatile metabolites and 40 volatile metabolites) were significantly different (Tables S3–S5). Multivariate statistical analyses based on the three different analytical instruments showed a clear separation among the three vinegars, implying that the raw materials affected the contents of both non-volatile and volatile metabolites in the vinegars (Figure 2A–C). Significantly different metabolites among the three types of vinegar were screened based on the VIP values (>0.7) and *p*-value (<0.05) derived from the respective PLS-DA score plots and one-way ANOVA. The relative content of 78 non-volatile and 54 volatile metabolites, which differed greatly depending on the raw material, are shown in Figure 2D,E.

In GV, amino acids, hydroxy fatty acids, and sugar alcohols from non-volatile metabolites, and aldehydes, pyrazines, and sulfides from volatile metabolites, were relatively abundant compared to the other two fruit vinegars. The abundant metabolites could originate and ferment from the raw materials. Brown rice, particularly rice bran, is a good source of amino acids and bioactive compounds (e.g., phenolic compounds) [39,40]. We speculated that the observed hydroxy fatty acids, 2-hydroxyisovaleric acid and 2-hydroxyisocaproic acid, might have been produced from valine and leucine, which have been reported as abundant amino acids in brown rice [41]. Volatile metabolites in GV are complex and composed of buttery, nutty, and fruity flavor compounds. For instance, compounds producing buttery and nutty flavors (e.g., 2,3-butanedione, isovaleraldehyde, and benzaldehyde) and metabolites eliciting sweet and fruity flavors (e.g., 3-methyl-1-butanol, phenethyl acetate, and isobutyl acetate) were relatively more abundant in GV than in fruit vinegars [42,43]. Moreover, alkylpyrazines (e.g., 2,3-dimethylpyrazine, trimethylpyrazine, and tetramethylpyrazine), contributing to nutty and toasty flavors, and sulfur-containing volatile compounds (e.g., dimethyl disulfide and dimethyl trisulfide), giving onion-like odors, were relatively more abundant in GV that might originate from rice protein [44].

PV contains higher amounts of organic acids, some amino acids, phenolic compounds and esters than GV and AV. Comparatively higher amounts of lysine, phenylalanine, and their derivatives (e.g., pipecolic acid and phenolic compounds) were observed in PV, which might originate or be fermented from raw materials by microorganisms [45]. Higher amounts of sweet and fruity flavor compounds, such as 2-butanol, ethyl propionate, and ethyl butyrate, were observed in PV [46].

In contrast, AV contains comparatively higher amounts of carbohydrates (e.g., mono-and di-saccharides) and flavonoids in non-volatile metabolites, alcohols, and some esters in volatile compounds. The observed flavonoids (e.g., phloridzin, hesperidin, and quercetin derivatives) might have originated from apples, particularly from the red skin. Hexyl acetate, pentyl butyrate, and 2-terpineol were the major volatile metabolites that produced sweet, floral, and fruity aromas in AV. According to the untargeted metabolome analysis results, the unique taste and flavor of each vinegar type can be depicted by the combination of various non-volatile and volatile metabolites. The relationship between the vinegars and their unique metabolites was also verified in the PLS-DA biplots (Figure S1A–C).

Subsequently, we estimated the fermentation pathways of the three vinegars prepared from different raw materials based on the metabolome analysis results (Figure S2). It showed a distinct metabolic pathway distribution according to the raw materials. In GV, metabolism associated with amino acids, hydroxy fatty acids, sugar alcohols, pyrazines, sulfides, and ketones may be activated. In fruit vinegars, metabolism related to phenolic compounds and volatile fatty acid esters were outstanding in PV, whereas, in AV, carbohydrates, long-chain fatty acids, flavonoids, and volatile esters metabolism were comparatively dominant.

### 3.2. Antioxidant Activity of Grain, Persimmon, and Apple Vinegars

**Figure 3:**
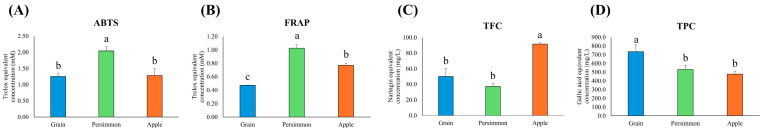
Results of antioxidant activities (**A**,**B**), total flavonoid content (TFC) (**C**), and total phenolic content (TPC) (**D**) in three types of vinegar. Different letters above the bars denote significant differences identified by Duncan’s multiple-range test (*p*-value < 0.05).

To compare the antioxidant activity of vinegars made from different raw materials, we conducted ABTS and FRAP assays and determined the TFC and TPC. Interestingly, PV exhibited statistically higher ABTS and FRAP assay results than GV and AV (Figure 3A,B). We guessed that gallic acid and pyrogallic acid, showing higher amounts in PV, may have contributed to the highest bioactive activity among the three vinegars in the ABTS and FRAP assays (Figure 2D and Figure 3A,B) [47,48]. AV and GV showed statistically higher TFC and TPC assay results, respectively, with similar antioxidant activities (Figure 3C,D). Integrating untargeted metabolomics with antioxidant assays, compounds such as methyldopahydrazine, 3,4-Dimethylprotocatechuic acid, salicylic acid, and 3-phenyllactic acid—particularly abundant in GV—may be responsible for the highest TPC and antioxidant activity (Figure 2D and Figure 3). Notably, 3,4-Dimethylprotocatechuic acid, salicylic acid, and 3-phenyllactic acid—detected in various foods—are widely recognized for their antioxidant and anti-inflammatory properties, as they scavenge free radicals and protect cells from oxidative stress [49,50,51,52]. The elevated TFC and antioxidant activity observed in AV could be attributed to compounds such as phlorizin, quercetin-3-rhamnose, quercetin-3-glucose and hesperidin, and chlorogenic acid, which are potent antioxidants found in fruits and vegetables [53,54,55,56,57]. Overall, antioxidants in vinegars mainly derive from raw ingredients rather than from fermentation-derived metabolites.

### 3.3. Metabolite Comparison in Individual Vinegar Samples

**Figure 4:**
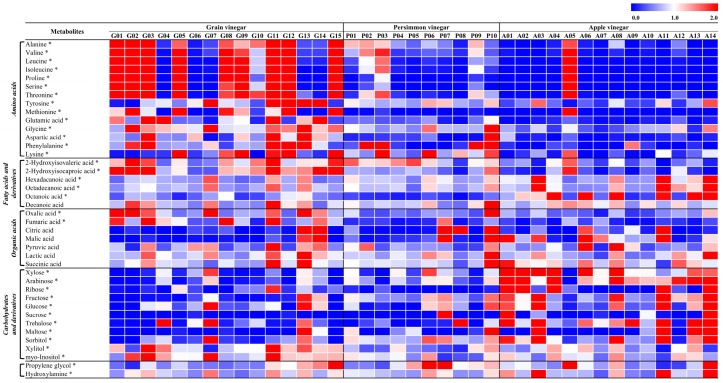
Heatmap of targeted metabolite analysis results on individual samples. All experiments were performed in triplicate. * The asterisks indicate the significant difference (VIP score > 0.7, *p*-value < 0.05).

To investigate the metabolite differences in each vinegar product manufactured by different producers, we performed metabolite analysis using 39 vinegar products using GC-TOF-MS and multivariate statistical analysis. Similar to the PLS-DA obtained from the pooled QC samples, the resultant partial least squares discriminant analysis (PLS-DA) using each product showed a separating pattern of the three vinegars, although each product fermented from the same main ingredient was scattered (Figure S3). Forty metabolites were tentatively identified, and their relative abundances are shown in Figure 4. The relative content of each metabolite was notably different depending on the vinegar product, even for the same type of vinegar. For example, G04, G06, G07, and G10 had relatively lower content of most detected amino acids than other GVs, while G13 and G14 contained comparatively lower amounts of most of amino acids but more glutamic acid, glycine, aspartic acid, and phenylalanine. Among PVs, P10 had different metabolite compositions, abundant fatty acids, organic acids, and carbohydrates compared to other PVs. Among the AVs, A05 showed a markedly distinct composition, containing higher amounts of amino acids but fewer carbohydrates than the other apple vinegars. Due to the variation in metabolites across individuals, the tastes and nutritional value of each product can differ, despite products belonging to the same vinegar category. We assume that the variation among individual products is caused by differences in ingredients, fermentation microorganisms, and fermentation conditions (e.g., temperature, duration).

### 3.4. Quantitative Analysis of Organic Acids, Amino Acids, and Fatty Acids

**Figure 5:**
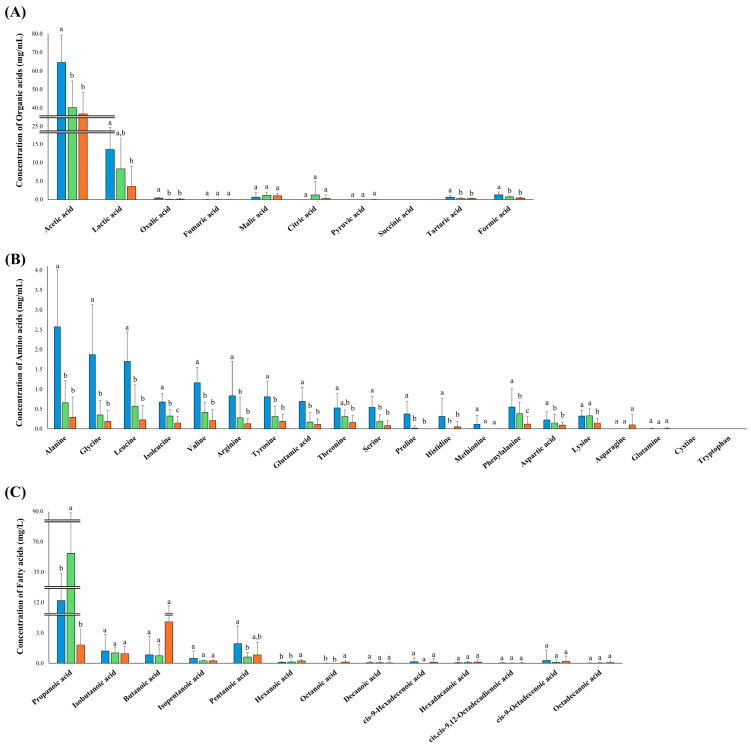
Quantification of organic acids (**A**), amino acids (**B**), and fatty acids (**C**) in GV (●), PV (●), and AV (●). Significant differences between groups are indicated by different letters above the bars based on Duncan’s multiple-range test (*p*-value < 0.05).

Next, we quantified the essential nutrients (e.g., organic acids, amino acids, and fatty acids) in the 39 vinegar products. For quantitative analysis, we listed the metabolites based on non-targeted metabolite analysis and previous studies that analyzed vinegars, resulting in 43 essential nutrients (Table S6). Quantitative analysis was conducted based on the standard curves of the listed metabolites (Table S2). The quantified results of each product are presented in Tables S7–S9. Furthermore, to investigate the amounts of metabolites in each type of product, the average amounts of each metabolite in the three types of vinegar are presented as bar graphs, with error bars representing the deviation of the metabolite concentrations within each product (Figure 5). The quantification data showed an obvious distinction of metabolites in the three types of vinegar and the differences in each vinegar product, leading to the unique taste and flavor properties that define each vinegar product. Moreover, we were able to figure out the major metabolites in each product through quantification.

The content of acetic acid, the key organic acid in vinegar, differed among the three types of vinegar. The highest average amount of acetic acid was observed in GV (ranging from 39.33 to 78.39 mg/mL), followed by PV (ranging from 21.94 to 58.00 mg/mL) and AV (ranging from 10.97 to 49.54 mg/mL). Lactic acid, the second most abundant organic acid in the vinegars, exhibited a similar pattern. The GV had higher lactic acid (from 3.95 to 38.22 mg/mL) content than PV (from 1.4 to 31.22 mg/mL) and AV (from 1.4 to 31.22 mg/mL). Additionally, oxalic acid (from 0.14 to 0.97 mg/mL), tartaric acid (from 0.23 to 1.87 mg/mL), and formic acid (from 0.63 to 3.87 mg/mL) are also present in statistically higher amounts in GV. Unlike other organic acids, the average citric acid content in PV was higher (LOD to 12.13 mg/mL) than that in GV and AV.

Most amino acids were significantly more abundant in GV than in PV and AV. Among the measured amino acids in GV, alanine (0.54 to 5.99 mg/mL) was the most abundant, followed by glycine (0.26 to 4.41 mg/mL) and leucine (0.58 to 2.81 mg/mL). Among fruit vinegars (PV and AV), PV contained more amino acids than AV, although the statistical differences in most amino acids between the two fruit vinegars were insignificant. The PV showed statistically higher amounts of isoleucine (0.19 to 0.72 mg/mL), phenylalanine (LOD to 1.03 mg/mL), and lysine (LOQ to 0.6 mg/mL) than AV. Asparagine was not detected in GV and PV and was only observed in four AV products (LOD to 1.00 mg/mL).

Most short-chain fatty acids containing fewer than six carbons were present in all types of vinegar. Propanoic acid was the most abundant among detected fatty acids and significantly higher in PV (5.88 to 100.67 mg/L), followed by GV (LOD to 92.13 mg/L) and AV (LOD to 5.28 mg/L). The content of butanoic acid (LOQ to 23.58), hexanoic acid (0.04 to 0.48 mg/L), and octanoic acid (LOD to 0.62 mg/L) were relatively higher in AV than in the other two vinegars. However, pentanoic acid was significantly more abundant in GV (LOD to 6.36 mg/L) than in AV (0.13 to 5.24 mg/L) and PV (0.12 to 1.48 mg/L).

The quantification result showed distinguished patterns not only among the three types of vinegar, but also in individual vinegar samples. Thus, we tried to compare the ingredients and chemical properties with those of the detected metabolites (Table S1). However, we were unable to determine the factors affecting metabolite differences in the same types of vinegar. We speculate that other factors not mentioned in the product information may have caused metabolite differences in each product, such as fermentation time, temperature, and microorganisms. It is necessary to analyze microbial communities during the fermentation process and investigate the relationship between microbial dynamics and metabolite changes in future studies. This study suggests that manufacturers can improve vinegar’s antioxidant content through careful raw material selection and refine flavor profiles by adjusting fermentation conditions, allowing consumers to select vinegar varieties based on their nutritional and organoleptic requirements.

## 4. Conclusions

This study’s comprehensive metabolite profiling highlights the profound diversity in nutrients, flavors, and bioactivities among grain, persimmon, and apple vinegars. Notably, our analysis identified over 130 metabolites, including amino acids, phenolics, organic acids, and volatiles, with significant variations aligned with raw ingredients and fermentation processes. The strong correlations observed between bioactive compounds—such as phenolic compounds and flavonoids—and antioxidant activity deepen our understanding of how these metabolites contribute to health benefits. From a practical perspective, these insights enable manufacturers to refine production processes and enhance product quality by targeting specific metabolites associated with health benefits and sensory attributes. Furthermore, this research enriches the knowledge of the chemical and bioactive components in commonly used vinegars, helping consumers make healthier selections and emphasizing the importance of making informed product choices. Overall, the findings provide a crucial foundation for future research, product development, and dietary recommendations aimed at health improvement through functional foods.

## Data Availability

The data of the research is available through the corresponding writer upon reasonable demand.

## Supplementary Materials

The following supporting information can be downloaded at: https://www.mdpi.com/article/10.3390/antiox14081029/s1, Figure S1: PLS biplots summarize the dominant metabolites distinguishing the three fermented vinegar types based on the untargeted metabolite analysis; Figure S2: Metabolic pathway and relative metabolite abundance of three types of vinegar. The metabolic pathway was adopted from the KEGG database. The pathways highlighted with blue, orange, and green backgrounds show abundant metabolites in GV, PV, and AV, respectively; Figure S3: Partial least squares discriminant analysis (PLS-DA) score plot of GV (●), PV (●), and AV (●) using individual samples; Table S1: Sample information of grain, persimmon, and apple vinegars; Table S2: Standard curve equations of organic acids, amino acids, and fatty acids used in the quantitative analysis of grain, persimmon, and apple vinegars; Table S3: Identified metabolites in grain, persimmon, and apple vinegars based on GC-TOF-MS analysis; Table S4: Identified metabolites in grain, persimmon, and apple vinegars based on UHPLC-Orbitrap-MS/MS analysis; Table S5: Identified metabolites in grain, persimmon, and apple vinegars based on HS-SPME-GC-TOF-MS analysis; Table S6: Organic acid, amino acid, and fatty acid list used in the quantitative analysis of grain, persimmon, and apple vinegars; Table S7: The concentration of organic acids, amino acids, and fatty acids in grain vinegars; Table S8: The concentration of organic acids, amino acids, and fatty acids in persimmon vinegars; Table S9: The concentration of organic acids, amino acids, and fatty acids in apple vinegars.
