# Supplementary figures and images for: Qualitative and Quantitative Metabolite Comparison of Grain, Persimmon, and Apple Vinegars with Antioxidant Activities
Source: Antioxidants (Basel). 2025 Aug 21;14(8):1029. doi: 10.3390/antiox14081029 (PMC12382752; doi:10.3390/antiox14081029)

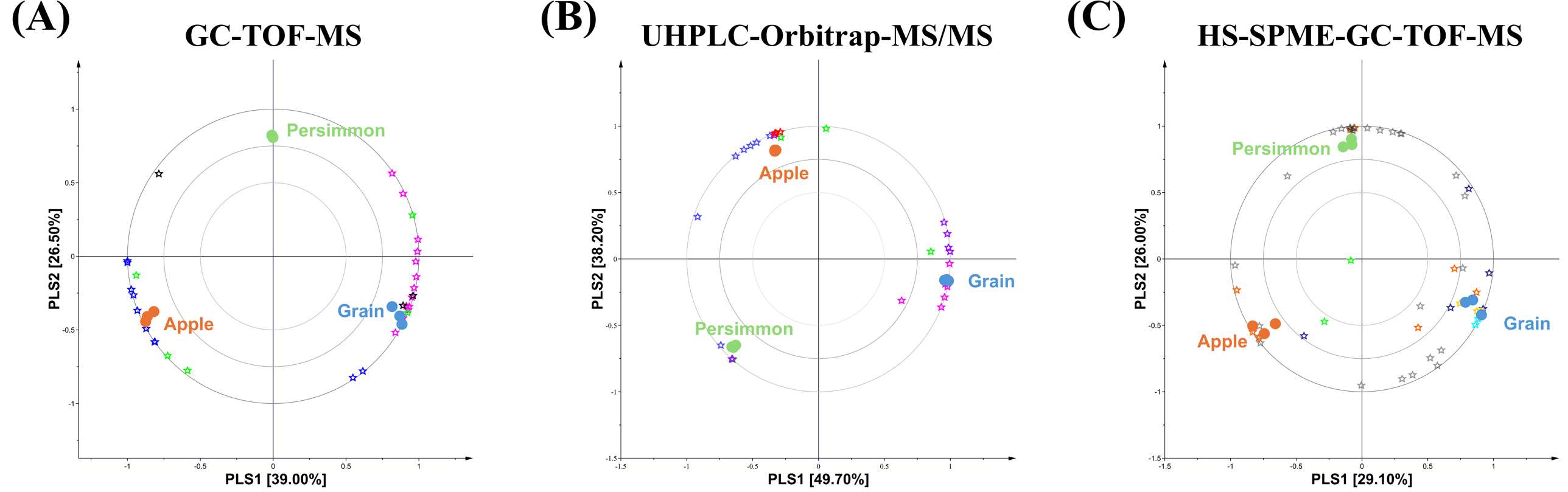

Supplement: Supplementary file 1 [file antioxidants-14-01029-s001.zip › Fig S1.tif]

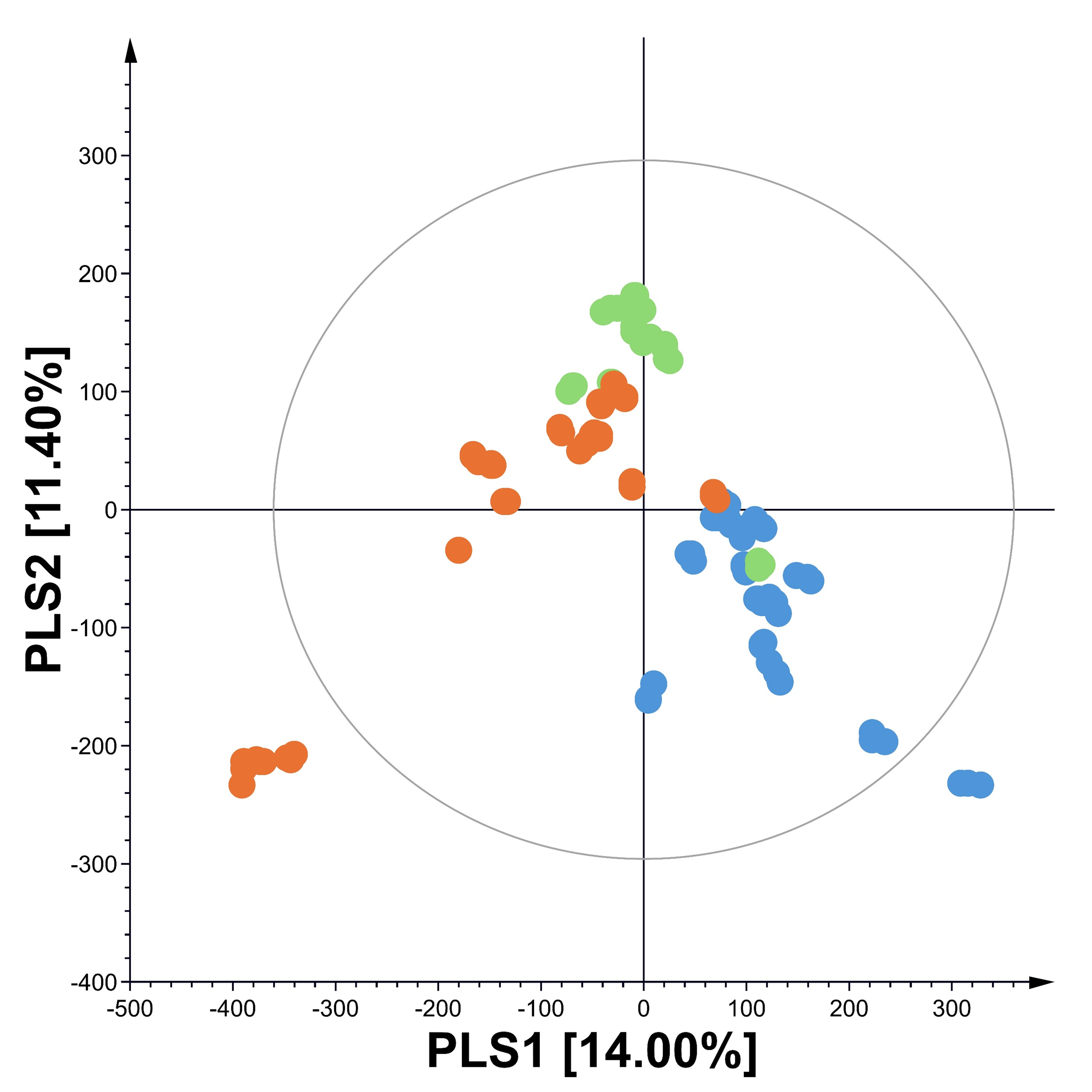

Supplement: Supplementary file 1 [file antioxidants-14-01029-s001.zip › Fig. S3.tif]
